# Lamellipodium extension and membrane ruffling require different SNARE-mediated trafficking pathways

**DOI:** 10.1186/1471-2121-11-62

**Published:** 2010-08-10

**Authors:** Michael Skalski, Qing Yi, Michelle J Kean, Dennis W Myers, Karla C Williams, Angela Burtnik, Marc G Coppolino

**Affiliations:** 1 Department of Molecular and Cellular Biology, University of Guleph, Guelph, ON N1G 2W1, Canada

## Abstract

**Background:**

Intracellular membrane traffic is an essential component of the membrane remodeling that supports lamellipodium extension during cell adhesion. The membrane trafficking pathways that contribute to cell adhesion have not been fully elucidated, but recent studies have implicated SNARE proteins. Here, the functions of several SNAREs (SNAP23, VAMP3, VAMP4 and syntaxin13) are characterized during the processes of cell spreading and membrane ruffling.

**Results:**

We report the first description of a SNARE complex, containing SNAP23, syntaxin13 and cellubrevin/VAMP3, that is induced by cell adhesion to an extracellular matrix. Impairing the function of the SNAREs in the complex using inhibitory SNARE domains disrupted the recycling endosome, impeded delivery of integrins to the cell surface, and reduced haptotactic cell migration and spreading. Blocking SNAP23 also inhibited the formation of PMA-stimulated, F-actin-rich membrane ruffles; however, membrane ruffle formation was not significantly altered by inhibition of VAMP3 or syntaxin13. In contrast, membrane ruffling, and not cell spreading, was sensitive to inhibition of two SNAREs within the biosynthetic secretory pathway, GS15 and VAMP4. Consistent with this, formation of a complex containing VAMP4 and SNAP23 was enhanced by treatment of cells with PMA. The results reveal a requirement for the function of a SNAP23-syntaxin13-VAMP3 complex in the formation of lamellipodia during cell adhesion and of a VAMP4-SNAP23-containing complex during PMA-induced membrane ruffling.

**Conclusions:**

Our findings suggest that different SNARE-mediated trafficking pathways support membrane remodeling during ECM-induced lamellipodium extension and PMA-induced ruffle formation, pointing to important mechanistic differences between these processes.

## Background

Cell adhesion to an extracellular matrix (ECM) is a fundamental feature of multicellular organisms and is a critical component of development, tissue function and homeostasis. Adhesive interactions between cells and ECM are complex, requiring the regulation of integrin localization and activity, reorganization of the actin cytoskeleton and localized membrane remodeling. After engagement of an ECM substrate, integrin-mediated biochemical signaling induces the actin-driven formation of Rac1-dependent lamellipodia and/or Cdc42-dependent filipodia [1]. A cell spreads as lamellipodia are extended and new adhesive contacts are made with the ECM substrate, stabilizing the cellular protrusions [2].

Integrin-mediated cell spreading is accompanied by dramatic alterations to the shape and content of the plasma membrane, particularly at sites where new cell-ECM contacts are formed. These alterations include the extension of the membrane (formation of a lamellipodium) and the enrichment of integrins and F-actin structures in or near the protrusion. Much evidence supports the notion that intracellular membrane trafficking is required for the remodeling of the plasma membrane as well as the redistribution of integrins that occurs during cell spreading. Studies have identified roles for the GTPases Arf6 [3,4], Rab4 [5,6], Rab11 [3,5], Rab5 [7,8], Rab8 [9] and dynamin [10] in cell adhesion. These GTPases operate within multi-step membrane trafficking pathways [11,12], which contribute to the transport of integrins between endosomal compartments as well as to and from the cell surface. The intracellular compartments involved in these trafficking pathways may thus serve as reservoirs of integrins for use in attachment of the extending edge as cells spread.

Within the membrane trafficking pathways involved in cell adhesion, how the final stages of the pathways are regulated is not well understood. Most known membrane traffic pathways culminate in SNARE (soluble NSF attachment protein receptor)-regulated membrane fusion events. Specifically, docking and membrane fusion are regulated by membrane bound SNAREs found on the vesicle (v-SNARE) and target (t-SNARE) membranes [13]. SNARE-mediated vesicular traffic is potentially involved in several aspects of integrin-mediated adhesion, including control of the intracellular compartmentalization of integrins and remodeling of the plasma membrane at sites of lamellipodium extension. Recent studies from our laboratory [14] and others' [15] have implicated the function of SNARE proteins in cell adhesion and migration. Specifically, we have determined that the activity of the endosomal SNARE cellubrevin/VAMP3 (vesicle-associated membrane protein-3) is required for normal trafficking of β1integrin to the plasma membrane during adhesion [16], and that a plasma membrane SNARE, SNAP23 (synaptosome-associated protein of 23 kDa), facilitates spreading of Chinese hamster ovary (CHO) cells on fibronectin (FN) [17]. These previous studies have not defined the relationship between the function of cellubrevin/VAMP3 and that of SNAP23, nor the role of these SNAREs in plasma membrane remodeling during cell adhesion.

In the present study, we report the first identification of a SNARE complex, SNAP23-syntaxin13-VAMP3, that forms in response to cell adhesion to ECM. Using ectopic expression of inhibitory SNARE domains in CHO-K1 cells, we demonstrate that the functions of VAMP3, syntaxin13 and SNAP23 are required for the formation of lamellipodia during cell adhesion and migration. We also present the novel finding that the SNAP23-syntaxin13-VAMP3 complex functions in the trafficking of β1 integrin from a sorting endosome to a Rab11-containing recycling compartment during the delivery of adhesive receptors to the plasma membrane. As well, an interaction between VAMP4 and SNAP23 was identified that was enhanced specifically during PMA-stimulated membrane ruffle formation. Inhibition of SNAP23, VAMP4 and the Golgi SNARE, GS15, but not VAMP3 nor syntaxin13, impaired the formation of PMA-induced F-actin-rich ruffles. The results suggest that cell adhesion is dependent upon SNARE function for ECM-induced integrin trafficking and lamellipodium extension, while a distinct SNARE-dependent pathway leading from the trans-Golgi network is required for the formation of PMA-induced dorsal membrane ruffles. Our findings point to mechanistic differences between lamellipodium extension and membrane ruffle formation that include different SNARE-regulated pathways.

## Results

### A SNAP23-syntaxin13-VAMP3 complex is induced in CHO cells attached to fibronectin

**Figure 1:**
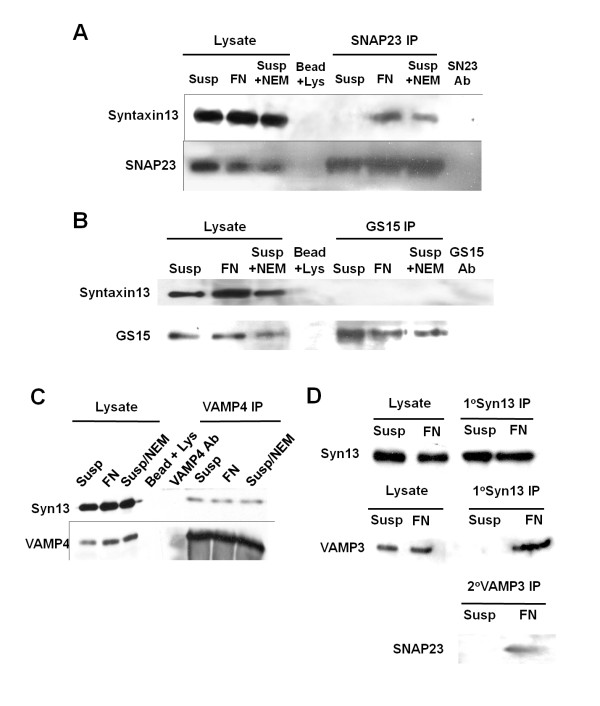
**SNAP23-syntaxin13-VAMP3 interaction in CHO cells is stimulated by adhesion to FN**. CHO-K1 cells were held in suspension after pretreatment with 1 mM NEM and 2 mM DTT (*Susp*), held in suspension after pretreatment with 1 mM NEM (*Susp + NEM*) or plated on FN (*FN*) for 20 min. The cells were lysed, and the indicated SNAREs were immunoprecipitated with antibodies coupled to sepharose beads from 1-1.5 mg of cellular protein. 10 μg aliquots of the lysates from which SNAREs were immunoprecipitated were run in 'Lysate' lanes. 'S13 Ab'/'SN23 Ab'/'GS15 Ab' indicate syntaxin13, SNAP23 and GS15 antibody control immunoprecipitations, where antibodies coupled to sepharose beads were incubated with lysis buffer, washed, and analyzed. (A) Syntaxin 13 immunoblot of SNAP23 immunoprecipitates showing immunoprecipitation of syntaxin 13 from cells plated on FN and cells treated with NEM, *upper blot*, and the same membrane reprobed for SNAP23, *lower blot*. (B) Syntaxin 13 immunoblot of GS15 immunoprecipitates, upper blot, and the same membrane reprobed for GS15, *lower blot*. (C) Syntaxin13 immunoblot of VAMP4 immunoprecipitates, and the same membrane reprobed for VAMP4, *lower blot*. (D) CHO-K1 cells, held in suspension (*Susp*) or plated on FN (*FN*), were lysed and syntaxin13 was immunoprecipitated with anti-syntaxin13 antibody. The syntaxin13 immunoprecipitates were washed and an aliquot (10%) of the eluate was analyzed by SDS-PAGE-western blot for syntaxin13, *upper blot*, and co-precipitated VAMP3, *middle blot*. The remainder of the syntaxin13-immunoprecipitate was re-suspended and immunoprecipitated with anti-VAMP3 antibody and then analyzed by SDS-PAGE-western blot for SNAP23, *lower blot*.

We have previously reported that the function of SNAREs, including SNAP23, are required for for endosomal trafficking and normal cell adhesion in CHO-K1 cells [16,17]. It has also been recently demonstrated that a SNARE complex comprised of SNAP25-syntaxin13-VAMP2 functions in endosomal trafficking in PC12 cells [18]. We therefore hypothesized that an analogous complex, comprised of SNAP23-syntaxin13-VAMP3, might function in cells of non-neuronal lineage during cell adhesion. To test this hypothesis, SNAP23 was immunoprecipitated from CHO-K1 cells held in suspension or plated on fibronectin (FN) and the immunoprecipitate was examined for associated syntaxin13. Syntaxin13 was more abundant in SNAP23 immunoprecipitate from cells attached to fibrontectin than from suspended cells (Fig. 1A). To confirm that the SNAP23-syntaxin13 interaction was NSF-sensitive, experiments were conducted using N-ethylmaleimide, an alkylating agent that inactivates NSF. Indeed, addition of NEM to cells held in suspension increased the amount of syntaxin13 found in SNAP23 immunoprecipitate (Fig. 1A). In contrast to the association of syntaxin13 with SNAP23, no syntaxin13 was co-precipitated with the Golgi SNARE GS15, in suspended cells, adherent cells or NEM-treated cells (Fig. 1B). Also in contrast to the interaction between SNAP23 and syntaxin13 found in adherent cells, was the fact that co-immonoprecipitation of syntaxin13 with VAMP4 was equally efficient in cells plated on FN, held in suspension or those treated with NEM (Fig. 1C). All immunoprecipitation experiments were independently repeated at least three times and the blots were scanned and analyzed by densitometry to quantify the levels of SNARE-binding partners in immunoprecipitates (Additional file 1, A and B). Normalized to the level of Syntaxin13 in SNAP23 immunoprecipitates from cells adherent to FN, the relative amount of syntaxin13 in SNAP23 immunoprecipitates from cells in suspension was 13.8 +/- 17.9% and from cells in suspension treated with NEM was 121.6 +/-24.0% (all results mean +/-SD)

In several cell types, including glial cells [19], platelets [20], and parotid acinar cells [21], VAMP3 and SNAP23 have been shown to be functional binding partners; thus, we predicted that VAMP3 may be part of the SNAP23-syntaxin13 complex that is formed during cell adhesion in CHO-K1 cells. To examine this possibility, sequential immunoprecipitation of syntaxin13 followed by VAMP3 was performed and the presence of SNAP23 analyzed. From cells held in suspension, little or no VAMP3 co-immunoprecipitated with syntaxin13 (Fig. 1D). This contrasted with the amount of VAMP3 detected in syntaxin13 immunoprecipitate from cells plated on FN (Fig. 1D). Subsequent immunoprecipitation of the primary syntaxin13 immunoprecipitate with VAMP3 antibody revealed the presence of SNAP23 (Fig. 1D), suggesting that in CHO-K1 cells a SNAP23-syntaxin13-VAMP3 complex forms, and the detection of this complex is enhanced by cellular adhesion to fibronectin.

To address the possibility that interactions amongst SNAREs were occurring post-lysis, 'lysate-mixing experiments' were performed. CHO cells were transfected with FLAG-SNAP23, or GFP-syntaxin13, or cotransfected with both these tagged constructs. Cotransfected cells were held in suspension or plated on FN for 20 min, lysed and the GFP-syntaxin13 was immunoprecipitated with anti-GFP antibody. Cells transfected with FLAG-SNAP23 or GFP-syntaxin13 alone were held in suspension and lysed. After lysis, the FLAG-SNAP23-containing and GFP-syntaxin13-containing lysates were mixed for 1 h followed by immunoprecipitation of the GFP-syntaxin13 using GFP antibody for 15 h. GFP immunoprecipitates were probed for the presence of FLAG-tagged SNAP23. FLAG-SNAP23 only co-precipitated with GFP-syntaxin13 in cells cotransfected with GFP-syntaxin13 and FLAG-SNAP23, not in samples where FLAG-SNAP23 and GFP-syntaxin13 were mixed *in vitro *(Additional file 2). Similar to the results seen in Fig. 1A, more SNAP23 co-precipitated with syntaxin13 from cells adherent to FN than from suspended cells. Collectively, these results indicate that VAMP3, syntaxin13 and SNAP23 specifically form an intracellular, NEM-sensitive complex in manner that is partly dependent upon cell adhesion.

### VAMP3, syntaxin13 and SNAP23 are required for lamellipodium extension

To assess the function of VAMP3, syntaxin13 and SNAP23 in cell spreading, we examined the effect of inhibiting these SNAREs during cell adhesion. SNAREs were inhibited by expressing GFP-tagged, truncated constructs of VAMP3 (aa 1-77; VAMP3cyto), VAMP4 (aa 1-118; VAMP4cyto), syntaxin13 (aa 1-250; syntaxin13cyto) and GS15 (aa 1-85; GS15cyto). These constructs lack transmembrane domains, and are therefore cytosolic proteins that can bind cognate SNAREs through their SNARE domain, putatively blocking interactions with membrane-anchored endogenous SNAREs and exerting a dominant-negative effect. A GFP-tagged SNAP23 construct with the C-terminal nine amino acids deleted was used (SNAP23^CΔ9^) to inhibit SNAP23 function. The mutant SNARE constructs described here have been used previously to inhibit membrane trafficking in several experimental systems [22-29]. To confirm the functionality of these constructs in the system used here, immunoprecipitates of endogenous cognate SNAREs were probed for both endogenous and ectopically-expressed, truncated SNAREs (Additional file 3). The truncated SNARE constructs were able to interact with endogenous SNAREs suggesting their ability to inhibit cognate binding partners within cells.

**Figure 2:**
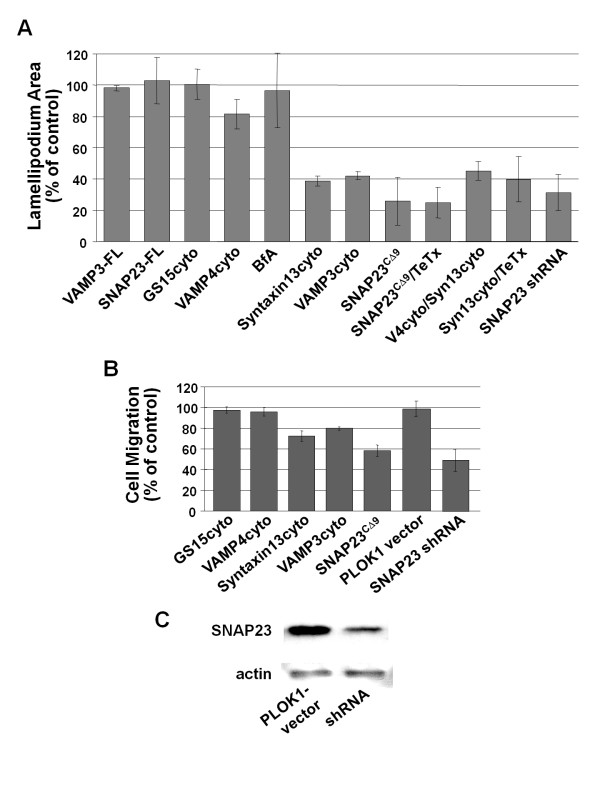
**Dominant-negative SNARE domains impair lamellipodium extension**. (A) CHO-K1 cells were transiently transfected with GFP-tagged constructs of the indicated SNAREs: GS15, VAMP4, VAMP3, syntaxin13 or SNAP23. Constructs encoded either the full length protein (FL) or the dominant-negative form (cyto; CΔ9 in the case of SNAP23). HeLa cells were transfected with a SNAP23 shRNA construct (SNAP23 shRNA). The cells were plated on FN-coated (20 μg/ml) coverslips and the extent of cell spreading was calculated as the increase in ventral surface area that occurred between 30 and 90 mins. The results are presented as percent of non-transfected control sample. More than 50 randomly selected transfected cells were analyzed per experiment. Means +/- SEM of at least three independent experiments are shown. (B) CHO cells were transfected as above, serum starved for 3 hrs and placed into a transwell chamber containing polycarbonate membranes coated on the underside with FN. Cells were allowed to migrate for 4.5 h, the membranes were fixed and the percentage of transfected cells migrated was assessed. Data is presented as the percentage of transfected cells migrated normalized to full-length controls and represent at least three independent experiments. (C) A sample of the same HeLa cells used in (A) were lysed and analyzed by Western blot for knockdown of SNAP23 expression. Actin served as a loading control. A set of representative blots are shown.

Lamellipodium extension in CHO cells was quantified as the increase in ventral cell area after attachment to FN (see material and methods). Similar to previous results using tetanus toxin light chain (TeTx-LC) to inhibit VAMP3 function [16], VAMP3cyto impaired lamellipodium extension by 58 ± 2% compared to untreated controls (Fig. 2A). Inhibiting syntaxin13 function by expression of syntaxin13cyto impaired lamellipodium extension by 61 ± 3% and expression of the truncated SNAP23^CΔ9 ^impaired lamellipodium extension by 75 ± 15% (Fig. 2A). None of the corresponding wild type (full length) SNARE proteins affected lamellipodium extension (VAMP3 and SNAP23 full length representative, all others not shown; Fig. 2A). Inhibition of VAMP4 or GS15 had a minimal effect on cellular spreading that was not statistically significant. Co-expression of tetanus toxin light chain - which cleaves VAMP3, impairs VAMP3-mediated traffic [30] and inhibits spreading in CHO cells [16] - together with SNAP23^CΔ9 ^or syntaxin13cyto did not result in further inhibition of cell spreading beyond that seen in cells expressing SNAP23^CΔ9 ^or syntaxin13cyto alone (Fig. 2A), nor did co-expression of syntaxin13cyto with SNAP23^CΔ9 ^(data not shown). Inhibition of VAMP4 combined with inhibition of syntaxin13 did not affect cell spreading more severely than inhibition of syntaxin13 alone (Fig. 2A). Similarly, no effect on cell spreading was observed when VAMP3 and VAMP4 were inhibited together in the same cells (data not shown). To confirm the involvement of SNAP23 in spreading, a short hairpin RNA (shRNA) construct designed to knock down expression of SNAP23 in human cells was transfected into HeLa cells. Transfection of HeLa cells with shRNA to SNAP23 inhibited spreading by 67 ± 12% (Fig. 2A). Knock down of SNAP23 was confirmed by Western blot (Fig. 2C). These findings suggest that VAMP3, syntaxin13 and SNAP23, but not VAMP4 or GS15, function in the same membrane trafficking pathway during lamellipodium extension.

To gain insight into the impact of SNARE inhibition on lamellipodium extension during directed cell migration, haptotactic transwell migration assays were performed. In these assays, cells in serum-free media were placed on top of a membrane with 8 μm pores and allowed to migrate through the pores toward FN coated on the underside of the membrane, as well as 10% serum in the lower chamber. Similar to the spreading assays, inhibition of GS15 and VAMP4 had little or no effect on migration, while syntaxin13cyto, VAMP3cyto and SNAP23^CΔ9 ^inhibited migration by approximately 30%, 20% and 40%, respectively (Fig 2B). Knock down of SNAP23 in HeLa cells, using shRNA as described above, also inhibited cell migration by approximately 50% (Fig. 2B).

**Figure 3:**
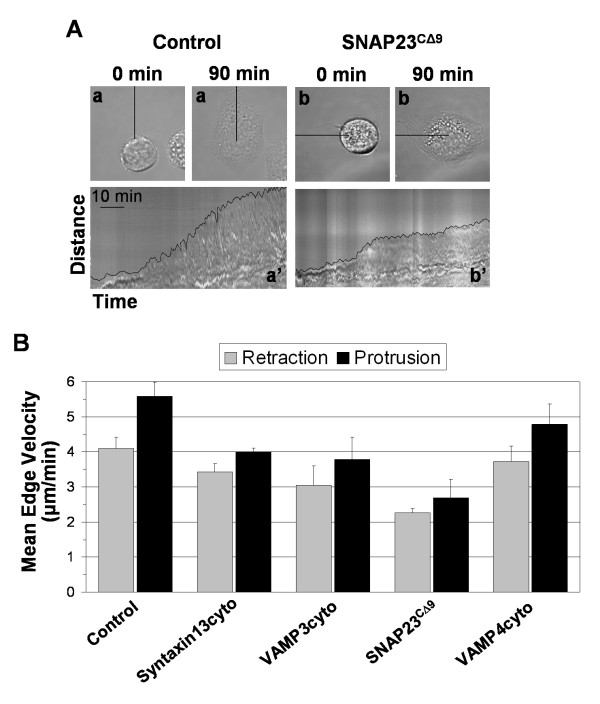
**Inhibition of SNAREs reduces the rate of lamellipodium protrusion**. Transfected CHO-K1 cells were plated in serum-free media on fibronetin for 90 min and monitored in real-time. (A) Representative control (a) and SNAP23^CΔ9 ^(b) cells are shown at initial acquisition (0 min) and after 90 min (90 min) in the top panels. Kymographs (a' and b') in lower panels represent progression of the cell edge along the lines indicated with the cell edge highlighted. (B) The protrusion and retraction rate of the cell edge was determined from kymographs using ImageJ software. Data is presented as the mean velocity of immediate edge movement from at least two cell edges from at least 3 cells (when possible the most protrusive regions of the cell were measured).

The observation that blocking VAMP3, SNAP23 or syntaxin13 function led to an impairment of lamellipodium extension is consistent with two possible explanations. It is possible that cells with impaired SNARE function: 1) cannot extend lamellipodia efficiently; or 2) can extend lamellipodia, but the lamellipodia cannot form stable adhesive contacts with the substratum and therefore are retracted. To assess these two possibilities, lamellipodium protrusion and retraction were examined in real time from cells plated on fibronection for 90 minutes. Kymographs of cellular spreading were generated, and the rate and duration of protrusions and retractions were quantified. Representative kymographs are shown in Fig. 3A and the data is summarized in Fig. 3B. In support of a model where SNARE inhibition impairs lamellipodium protrusion, it was found that cells expressing syntaxin13cyto, VAMP3cyto and SNAP23^CΔ9 ^did form lamellipodia, but did so at a significantly slower rate than control cells (p ≤ 0.05). Lamellipodia from control cells extended at a mean rate of 5.6 ± 0.4 μm/min, while syntaxin13cyto and VAMP3cyto cells protruded at a mean rate of 4.0 ± 0.1 and 3.8 ± 0.6 μm/min, respectively. Similar to the spreading assay above, SNAP23^CΔ9 ^proved to be the most potent inhibitor of extension, with SNAP23^CΔ9 ^cells protruding at a mean rate of 2.7 ± 0.5 μm/min, and VAMP4cyto only had a modest effect (mean protrusion rate of 4.8 ± 0.6 μm/min). SNARE inhibition also inhibited retraction velocity compared to control cells, though the differences where not as substantial as seen with protrusion velocity (Fig. 3B).

### Inhibiting VAMP3, syntaxin13 or SNAP23 alters trafficking of β1-integrin

**Figure 4:**
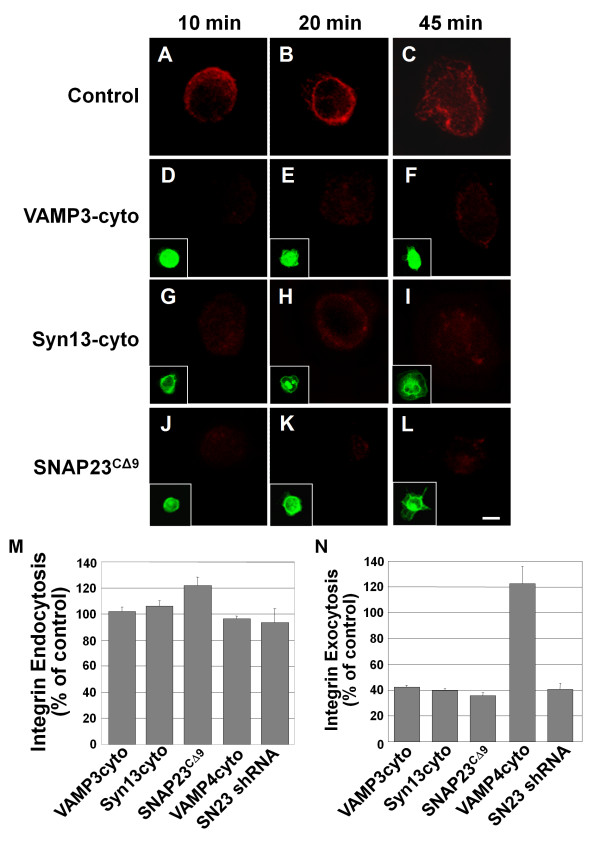
**Dominant-negative SNARE domains inhibit β1 integrin exocytosis**. CHO-K1 cells were transiently transfected for 21 h with GFP-tagged constructs of truncated syntaxin13 (Syn13cyto)[D-F], VAMP3 (VAMP3cyto)[G-I], VAMP4 (VAMP4cyto) or SNAP23 (SNAP23^CΔ9 ^- 15 h transfection) [J-L]. Untransfected control cells are shown in A-C. HeLa cells were transfected with a SNAP23 shRNA construct (SN23 shRNA) for 72 h. Cells were incubated with β1 integrin antibody in serum free media for 3 h to allowing internalization of the label. Then the cells were lifted with trypsin, removing remaining surface label, and were plated on 20 μg/mL FN for 10 min (A, D, G, J,), 20 min (B, E, H, K,) or 45 min (C, F, I, L). For exocytosis assays, cells were fixed and any surface exposed labeled-integrin was stained with AlexaFluor 594 secondary antibody (A-L, data for 20 min of adhesion shown in N). Insets show expression of GFP-tagged constructs (D-L). Images are 3 D reconstructions of a z-series. Scale bar represents 10 μm. (M and N) Integrin immunofluorescent staining at 20 min. was quantified in micrographs using ImageJ software. (M) To assess endocytosis of labeled integrin, cells were permeablized with 0.2% Trition X-100 in PBS before staining with AlexaFluor 594 secondary antibody (percent of non-transfected control). (N) Labeled integrin detected on the cell surface in non-permeabilized cells (percent of non-transfected control). Means ± SEM from three independent experiments (at least 20 cells per experiment) are shown in M and N.

In previous studies, we have demonstrated, using TeTx-LC, that VAMP3 is required for trafficking of β1 integrin from the recycling endosome to the plasma membrane and that this inhibition in traffic is a possible explanation for reduced spreading [16]. Here, we examined whether inhibition with the syntaxin13cyto, VAMP3cyto, SNAP23^CΔ9^, VAMP4cyto and the SNAP23 shRNA constructs also prevents exocytosis of β1 integrin. Integrin exocytosis was monitored by allowing internalized antibody-labeled β1 integrin to traffic to the plasma membrane during adhesion to FN. Internalization of label was confirmed by permeablization and staining with fluorescently labeled secondary antibody. Inhibiting SNARE function with the SNARE constructs or shRNA did not inhibit endocytosis of labeled integrin relative to controls (Fig. 4M). In non-transfected control cells, at 10 min, β1 integrin was visible on the cell surface and became more prominent at 20 min (Fig. 4A and 4B, respectively). At 45 min, the β1 integrin signal became more diffuse as the integrin was redistributed on the cell surface as well as internalized again (Fig. 4C). Transfection of cells with empty vector did not alter integrin trafficking (data not shown). Consistent with previous experiments using TeTx-LC [16], at 10 min VAMP3cyto-expressing cells show no labeled β1 integrin on their surface (Fig. 4D). After 20 and 45 min a modest amount of integrin is visible (Fig. 4E and 4F, respectively). In cells expressing syntaxin13cyto, little internalized β1 integrin is delivered to the cell surface at 10 min (Fig. 4G), while at 20 and 45 min time points only a modest amount of integrin has reached the surface (Fig. 4H and 4I, respectively). Cells expressing SNAP23^CΔ9 ^show little or no labeled integrin on their surface at all time points examined (Fig. 4J-L). The surface integrin staining was quantified for the 20 min time point of three independent experiments and is shown in Fig. 4N. When VAMP3, syntaxin13 or SNAP23 were inhibited the exocytosis of labeled integrin was decreased by 60%. Similar results were seen when SNAP23 was knocked-down using an shRNA construct (Fig. 4N). By contrast, expression of VAMP4cyto slightly increased the appearance of label at the surface (Fig. 4N).

**Figure 5:**
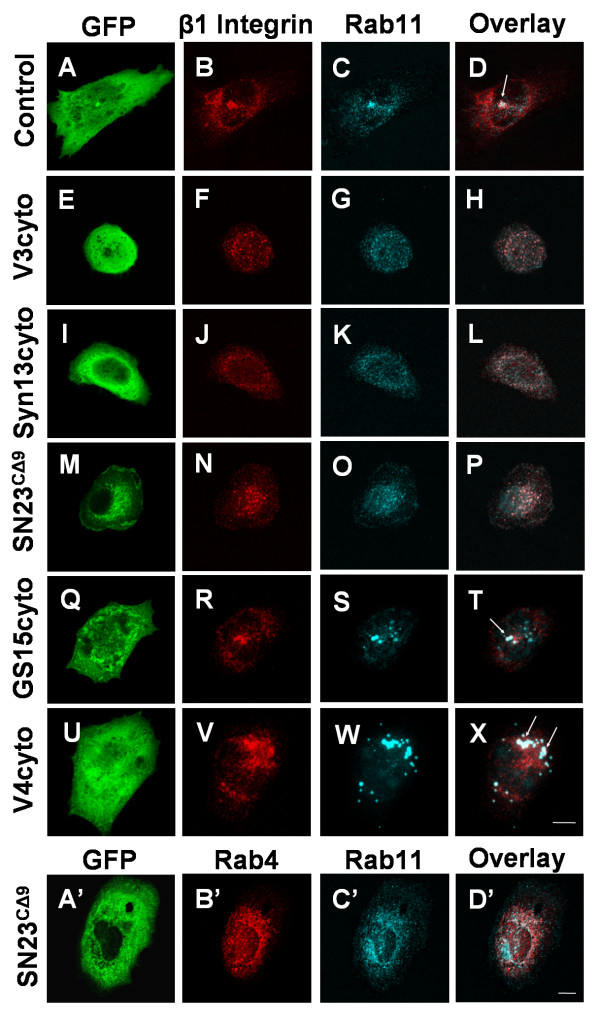
**Dominant-negative SNARE domains disrupt β_1 _integrin trafficking into the recycling endosome**. CHO-K1 cells were transiently transfected for 22 h with GFP [A-D] or GFP-tagged constructs of truncated VAMP3 (V3cyto) [E-H], syntaxin13 (Syn13cyto) [I-L], SNAP23 (SNAP23^CΔ9 ^- 15 h transfection) [M-P, A'-D'], GS15 (GS15cyto) [Q-T], or VAMP4 (V4cyto) [U-X]. Cells were labeled with anti-β1 integrin antibody in serum free medium for 1 h to allow internalization of integrin-antibody complexes. Cells were then washed in 0.2 M glycine, pH 2.5, fixed, and stained for Rab11 [C, G, K, O, S, W and C'] and the intracellular integrin-antibody complexes [B, F, J, N, R, and V], or Rab4 [B']. Overlays of the Rab11 and β1 integrin staining are shown in D, H, L, P, T and X, and Rab4 and Rab11 overlays are shown in D'. Arrows indicate conventional colocalization of β1 integrin in a central Rab11-containing compartment. Images are 3 D reconstructions of a z-series. Scale bar represents 10 μm.

Having shown that VAMP3, syntaxin13 and SNAP23 form a complex, and that inhibition of these SNAREs impairs integrin exocytosis, we sought to determine where within the cell β1 integrin was being trapped. CHO cells expressing dominant-negative SNARE domains were incubated with β1 integrin antibody for one hour followed by an acid wash to remove surface antibody. The subcellular localization of the internalized integrin and the endocytic compartment markers Rab11 and Rab4 was then assessed (Fig. 5 and Additional file 4). In control, GFP-transfected cells (Fig. 5A-D), a portion of the internalized integrin localizes in the cell periphery with the sorting endosome marker Rab4 (Additional file 4B-D). Another portion of β1 localizes in a concentrated, perinuclear compartment, overlapping with the recycling endosome marker Rab11 (Fig. 5B-D, see arrow in 5D). These distributions were also observed in non-transfected controls (data not shown). Strikingly, in cells expressing VAMP3cyto, syntaxin13cyto or SNAP23^CΔ9^, the peripheral localization of β1 was unaltered, but there was little or no perinuclear β1- or Rab11-containing compartment (Fig. 5E-P). In cells expressing the dominant-negative SNARE inhibitors, the concentrated, central compartment containing Rab11 appeared to be disrupted, becoming punctate and peripherally distributed (Fig. 5E-P, contrast with Fig. 5B-D). It is important to note, however, that the association of integrin with Rab11 was maintained, though the morphology and location of this compartment was dramatically altered. Further analysis revealed that expression of the mutant SNAREs had caused Rab11 to redistribute and extensively co-localize with the Rab4 compartment (Fig. 5A'-D'; SNAP23^CΔ9 ^shown as representative images). The inhibition of this pathway does not appear to be specific to integrin trafficking as the VAMP3cyto, syntaxin13cyto and SNAP23^CΔ9 ^also inhibit transferrin trafficking into a perinuclear compartment (Additional file 5). Expression of VAMP4cyto (Fig. 5Q-T) and GS15cyto (Fig. 5U-X) had no effect on β1 integrin or transferrin (Additional file 5G-H) trafficking into the perinuclear compartment. Neither VAMP4cyto nor GS15cyto altered the morphology of the recycling endosome to the degree that VAMP3cyto, syntaxin13cyto or SNAP23^CΔ9 ^did (Fig. 5S and 5W).

**Figure 6:**
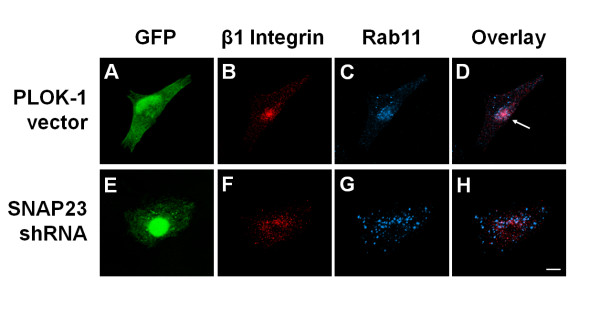
**shRNA-mediated downregulation of SNAP23 disrupts β_1 _integrin trafficking into the recycling endosome**. Hela cells were transiently transfected for 72 h with GFP + PLOK-1 vectror [A-D] or GFP + vector containing SNAP23-targeting shRNA [E-H]. Cells were labeled with anti-β1 integrin antibody in serum free media for 1 h to allow internalization of integrin-antibody complexes. Cells were then washed in 0.2 M glycine, pH 2.5, fixed, and stained for the intracellular integrin-antibody complexes [B, F] and Rab11 [C, G]. Overlays of the Rab11 and β1 integrin staining are shown in D and H. Arrows indicate conventional colocalization of β1 integrin in a central Rab11-containing compartment. Images are 3 D reconstructions of a z-series. Scale bar represents 10 μm.

To further assess the role of SNAP23 in trafficking of β1 integrin, we monitored trafficking of the integrin in HeLa cells expressing shRNA to reduce expression of SNAP23 (as described for Fig. 2). HeLa cells transfected with control PLOK-1 vector had similar intracellular distributions of β1 and Rab11 to those described above, with a population of these markers co-localizing in a concentrated, perinuclear compartment (Fig. 6A-D, see arrow in 6D). Consistent with cells expressing SNAP23^CΔ9^, HeLa cells transfected with SNAP23 shRNA showed little or no perinuclear β1- or Rab11-containing compartment (Fig. 6F-H). Taken together, these data suggest that inhibition of VAMP3, syntaxin13 or SNAP23 disrupts trafficking from the sorting endosome to the recycling endosome, resulting in perturbed recycling of integrin β1.

### SNAP23 and VAMP4 function are required for PMA-induced membrane ruffling

The observation that blocking VAMP3, SNAP23 or syntaxin13 function inhibits integrin trafficking suggested that SNARE-mediated trafficking supports lamellipodium extension by providing ECM receptors at the leading edge to aid in attachment and stabilization of lamellipodia. Previous studies, however, have shown that when membrane trafficking is inhibited integrin-mediated cell attachment is not altered [16], and measurements herein suggest that lamellipodium protrusion was inhibited rather than attachment (Fig. 3A and 3B). These results are consistent with the notion that SNARE function is required for the formation of actin-based membrane protrusions. To determine if inhibition of SNARE-mediated traffic perturbs actin-based membrane remodeling independently of cell attachment, we tested the ability of CHO-K1 cells to form F-actin containing ruffles in response to PMA. This was used as an indicator of the cells' capacity to form membrane protrusions without any requirement for the protrusions to attach to the underlying substratum.

**Figure 7:**
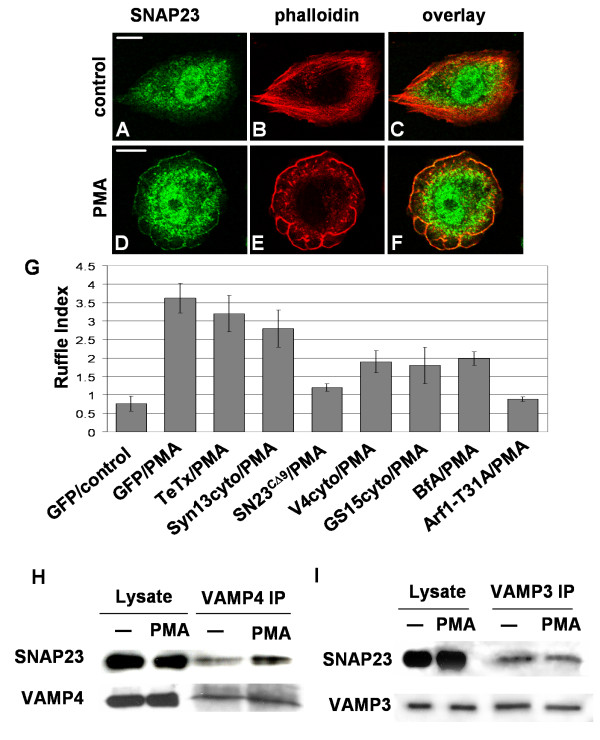
**Analysis of SNARE function in PMA-induced membrane ruffle formation**. CHO-K1 cells were treated with 500 nM PMA in DMEM (PMA) or DMEM alone (control) for 10 mins, fixed, permeabilized and stained with anti-SNAP23 antibody, Alexa-488-conjugated secondary antibody (A, D) and rhodamine-phalloidin (B, E). A-C, control cell; D-F, PMA-treated cell. Some cross-reactive binding of the SNAP23 antibody in the nucleus is observed (A, D). C and F are overlays of panels A and B and D and E, respectively. Scale bar represents 10 μm. (G) CHO-K1 cells were transfected with GFP vector alone, GFP-tagged dominant-negative constructs of the indicated SNAREs, or GFP together with the light chain of tetanus toxin. HeLa cells were transfected for 72 h with GFP + PLOK-1 or GFP + vector containing SNAP23-targeting shRNA. Cells were treated with 500 nM PMA in DMEM (PMA) or DMEM alone (control) for 10 mins, fixed, permeabilized and stained with rhodamine-phalloidin. Samples were then imaged by confocal microscopy and actin-rich ruffles on the dorsal region of the cells were quantified as described previously. Ruffle indices (expressed as mean +/- SEM) are shown from three independent experiments (more than 25 randomly selected transfected cells were analyzed per condition in each experiment). (H and I) CHO-K1 cells were treated as above, lysed, and VAMP4 (H) or VAMP3 (I) were immunoprecipitated. Immunoprecipitates were washed and analyzed by SDS-PAGE-western blotting for SNAP23, *upper blots*. Then membranes were stripped and reprobed for the precipitated SNARE, *lower blots*.

In CHO cells, endogenous SNAP23 was detected in PMA-induced membrane ruffles (Fig. 7A-F). While this observation is consistent with a role for SNAP23 in ruffle formation, we sought to directly assess the function of SNAP23, and SNAREs with which it interacts, in the formation of ruffles. To quantify membrane ruffles, we have developed a novel and rigorous method to automatically define and measure F-actin-rich ruffle structures in confocal micrographs [31]. CHO cells were transfected with the inhibitory SNARE constructs described above and F-actin-based ruffle formation was quantified. As seen in Fig. 7G, expression of SNAP23^CΔ9 ^substantially reduced ruffle formation - the ruffle index for GFP-expressing/PMA-treated cells was 3.62 ± 0.4 and that for SNAP23^CΔ9^-expressing/PMA-treated cells was 1.20 ± 0.21, a 67% decrease (Fig. 7G). Confirming the effect of SNAP23 inhibition on membrane ruffling, we observed that shRNA mediated knock-down of SNAP23 expression reduced ruffle formation in HeLa cells (ruffle index of 1.57 ± 0.3, compared to PLOK1 vector controls with a ruffle index of 3.51 ± 0.47). HeLa cells were used for these experiments due to their compatibility with our human sequence-specific shRNA construct.

Inhibition of VAMP3 or syntaxin13 did not significantly alter PMA-induced membrane ruffle formation (Fig. 7G). Results from transfection of either VAMP3cyto (data not shown) or TeTx light chain (Fig. 7G), equally indicated a lack of effect on membrane ruffling. Inhibition of syntaxin13 caused an observable reduction in ruffle formation but this was not statistically significant compared to GFP-transfected/PMA-treated control (p = 0.19, Student's t-test). In contrast with their effect on cell spreading, inhibition of VAMP4 and GS15 caused a 50% reduction in the formation of PMA-induced ruffles (Fig. 7G). Since GS15 [28] and VAMP4 [23] function in the biosynthetic-secretory membrane trafficking pathway, brefeldin A and a dominant-negative Arf1 mutant (Arf1-T31A), known inhibitors of the biosynthetic-secretory pathway [32,33], where used to confirm the involvement of this membrane trafficking pathway in PMA-induced ruffling. Brefeldin A and Arf1-T31A reduced ruffles by 45% and 75%, respectively (Fig. 7G).

The observation that VAMP4cyto, SNAP23^CΔ9 ^inhibited membrane ruffling suggested that these SNAREs may function in the same ruffling-dependent trafficking pathway. Therefore, we examined whether PMA could induce the formation of a VAMP4-SNAP23 complex in serum starved CHO cells. Stimulation with PMA increased the amount of SNAP23 that immunoprecipitated with VAMP4 (Fig. 7H), while having no effect on the amount of SNAP23 that co-precipitated with VAMP3 (Fig. 7I). As in Fig. 1, the amounts of SNARE-binding partners in the immunoprecipitates were quantified from at least three independent experiments by densitometric analysis of Western blots (see Additional file 1C and 1D). Normalized to the level of SNAP23 in VAMP4 immunoprecipitates from non-induced control cells, the relative amount of SNAP23 inVAMP4 immunoprecipitates from cells induced with PMA was 160.1 +/- 41.3%. Normalized to the level of SNAP23 in VAMP3 immunoprecipitates from non-induced control cells, the relative amount of SNAP23 inVAMP3 immunoprecipitates from cells induced with PMA was 90 +/- 8.4%. The results suggest that PMA specifically induces a SNAP23-VAMP4 complex during the formation of membrane ruffles.

## Discussion

In the present study, we have shown that VAMP3, syntaxin13 and SNAP23 form a complex during CHO cell adhesion to FN. While previous studies have documented the existence of a VAMP3-SNAP23-containing SNARE complex [34], herein is the first evidence that formation of such a complex can be stimulated by cell-ECM interaction. The data are also the first to implicate the function of the endosomal SNARE syntaxin13 in cell adhesion and migration. Inhibition of syntaxin13 impaired cell spreading and migration to an extent that was quantitatively similar to the impairment caused by inhibition of VAMP3 and the effects of inhibiting VAMP3 and syntaxin13 during cell spreading were not additive, suggesting that these SNAREs function along the same trafficking pathway. This conclusion is consistent with our detection of SNAP23 in association with VAMP3 that had been immunoprecipitated from syntaxin13 immunoprecipitate (Fig. 1D). Sequential immunoprecipitation of endogenous proteins is not an efficient process, often providing poor protein recovery, and detection of these interactions here, in response to cell-ECM interaction, is an important observation. Furthermore, evidence of the VAMP3-syntaxin13-SNAP23 complex was obtained through analysis of both endogenous proteins (Fig. 1) and ectopically expressed constructs (Additional file 3), and in both cases the SNARE interactions were enhanced by cell adhesion to ECM substrate.

The cellular importance of the SNAP23-syntaxin13-VAMP3 complex is revealed by data showing that cell spreading and β1 integrin exocytosis were impaired when the function of these SNAREs was disrupted. Our findings are consistent with much evidence indicating that cellular adhesion requires regulated membrane traffic, including the recycling of integrins and the redistribution of integrins to sites of lamellipodium protrusion [3,5,6,35]. Recent studies directly implicate VAMP3 in trafficking of integrins during CHO cell adhesion [16] and SNAP23 in focal adhesion formation [17]. In the present study, we now report that inhibiting SNAP23 reduced cell spreading by approximately 75%, whether VAMP3 was inhibited or not, consistent with a model in which the functions of these two SNAREs during cell adhesion are part of a common pathway. Overall, these results argue that the SNAP23-syntaxin13-VAMP3 interaction is functionally important to this cell-ECM interaction.

Our findings do not exclude the possibility of other SNAREs being involved in the formation of lamellipodia, but they do suggest that SNAP23 function may be central in this process. It is possible that SNAP23 functions at the plasma membrane and at internal endosomes to facilitate the delivery of secreted and/or recycled components during lamellipodium extension. Other SNAREs (e.g. v-SNAREs in endosomal compartments) may function in compartment-specific pathways, but all these pathways may, in turn, depend on SNAP23 function. If so, then SNAP23^CΔ9 ^would be expected to be a more potent inhibitor of cell motility than other soluble SNARE domains, as was observed. In support of this model, others have reported that a VAMP2-syntaxin13-SNAP25 complex mediates traffic from a sorting endosome to a recycling endosome in neurons [18,36]. In non-neuronal cells, a similar complex, such as the VAMP3-syntaxin13-SNAP23 complex described here, may be involved in an analogous trafficking pathway. Our findings confirm that blocking syntaxin13, VAMP3 or SNAP23 function interrupts β1 integrin and transferrin trafficking to a perinuclear recycling compartment, causing extensive colocalization with Rab4 (a sorting endosome marker; Additional file 4). These results are the first demonstration of a SNARE complex that functions in transport from a sorting endosome to a recycling endosome in non-neuronal cells.

The effects of SNARE inhibition on cell spreading and migration suggest that SNARE function is required to support the localized extension of the plasma membrane to form lamellipodia. It is interesting to note that, compared to the effects on lamellipodium extension (Fig. 2A), relatively modest effects were observed in cell migration (Fig. 2B). This may be explained by the fact that the cells do retain some capacity to form protrusions, albeit at a reduced rate (see Fig. 3B), and this impaired protrusive capacity supports some degree of cell migration while having a more negative impact on cell spreading. The results of live cell imaging, presented in Fig. 3B, reveal that when the function of syntaxin13, VAMP3 or SNAP23 is disrupted lamellipodia do form, but the protrusion velocity is significantly reduced. It has been demonstrated that protrusion velocity is dependent on dynamic actin polymerization at the edge of lamellipodia, independent of integrin function [37,38]. Here, we present evidence that PMA-induced F-actin dependent membrane ruffle formation was impaired by blocking SNAP23. Since membrane ruffle formation is not dependent on the attachment of membrane protrusions to a substratum, these results lead to the conclusion that the function of SNAP23 is required for the remodeling of the plasma membrane that is the basis for lamellipodium formation. In this context, SNARE-mediated traffic may be necessary for the insertion of membrane, from intracellular compartments, into the plasma membrane, or the trafficking of actin regulating proteins to the leading edge. It is also possible that other plasma membrane SNAREs (syntaxins 2-4) are involved in remodeling the plasma membrane. For example, syntaxin3 has been shown to be involved in cell membrane expansion [39]. The cells studied here do express syntaxin4 and we are currently developing assays to examine the function syntaxin4 and other SNAREs in cell membrane remodelling events.

The fact that inhibition of VAMP4 or GS15 impaired membrane ruffle formation is consistent with a previous study that suggested PMA-induced ruffle formation is dependent on Arf1 activity [40]. Arf1 is a well known regulator of the biosynthetic-secretory pathway and has also been ascribed important signaling functions leading to actin remodeling [41,42]. Given the results presented here, it is likely that VAMP4 and GS15 function in the secretory pathway along with Arf1. GS15 localizes to the Golgi, and GS15 function has been linked to retrograde traffic within the Golgi and trans-Golgi network [28,43]. VAMP4 is known to mediate retrograde traffic from endosomes and secretroy granules to the TGN [23,44-47], and has been implicated in GLUT4 anterograde traffic from the Golgi [48]. VAMP4 may also be directly involved in trafficking to the plasma membrane as a minor amount of VAMP4 is found at the plasma membrane [our unpublished data and [23]]. In the present study, our observations indicate that the functions of VAMP4 and GS15 are required for membrane ruffle formation in response to PMA, but the activity of these SNAREs is not necessary for ECM-stimulated lamellipodium extension. Conversely, the functions of VAMP3 and syntaxin13 are required for the efficient formation of lamellipodia during cell spreading on ECM, but not PMA-induced membrane ruffling. Therefore, these data indicate that SNARE-mediated membrane traffic is important for both lamellipodium extension and membrane ruffle formation, but that the SNARE-mediated pathways involved in these two processes are not identical.

## Conclusions

The results described here are the first to directly demonstrate that formation of a SNAP23-syntaxin13-VAMP3 complex is modulated during cell-ECM adhesion. The observations contribute to a growing body of evidence describing an important role for SNARE-mediated membrane traffic in cell adhesion. Importantly, the present findings advance our understanding of the role of SNARE-mediated membrane traffic in cell adhesion; it supports transport from a sorting endosome to a recycling compartment during the delivery of adhesive receptors to the plasma membrane. Furthermore, blocking the function of SNAP23 impaired the formation of membrane ruffles in response to PMA, a process that is expected to be independent of adhesive receptor localization. Thus, SNARE-mediated traffic may be needed to deliver membrane components that allow the plasma membrane to be remodeled during the elaboration of membrane ruffles, as well as lamellipodia. The fact that different SNAREs are required for the formation of ruffles and lamellipodia suggests that these processes have partially separate molecular mechanisms. We have now identified a SNARE complex that is induced in cells in response to ECM attachment and that is essential for normal cell adhesion. Future work to elucidate how SNARE complex formation is regulated during cell adhesion will further advance the understanding of cell-ECM interactions.

## Methods

### Reagents and cDNA constructs

All chemicals were purchased from Sigma Chemical Co. (St. Louis, MO) or Fisher Scientific (Nepean, ON) unless otherwise indicated. Antibodies against Rab4 and Rab11 were obtained from Cedarlane Laboratories Ltd. (Mississauga, ON) and BD Biosciences (Mississauga, ON), respectively. All secondary antibodies, rhodamine-labeled transferrin and rhodamine-labeled phalloidin were purchased from Invitrogen (Mississauga, ON).

The pcDNA3.1-SNAP23fl, pcDNA3.1-SNAP23^CΔ9^, pcDNA3.1-tetanus toxin light chain (TeTx-LC) and peGFP-VAMP3 were kind gifts from Dr. W.S. Trimble (Hospital for Sick Children, Toronto). The pEGFP-SNAP23^CΔ9 ^and pEGFP-SNAP23fl constructs were generated by inserting the *XhoI*/*EcoRI *fragment from the full length and truncated pcDNA3.1-SNAP23, respectively, into peGFP-C1. The FLAG-SNAP23 construct was generated by subcloning the full length SNAP23 into pcDNA3.1-FLAG, a kind gift from Dr. Nina Jones (University of Guelph, Guelph, Canada). The C-terminal enhanced green fluorescent protein (EGFP) constructs encoding wild-type syntaxin13, GS15 and VAMP4 were prepared by PCR amplification from a HeLa cell cDNA library and cloned into pEGFP-N1. The following oligonucleotides were used as primers: Syn13FOR (5'-CTAGCTCGA-GATGTATCGGAATCCCGGG-3') and Syn13REVcyto (5'-CTAGGAATTCGGACAAAGCACGAGGATACACA-3'), V3FOR (5'-CTAGCTCGAGATGTCTACAGGGGTGCCTTC-3') and V3REVcyto (5'-CTAGGAATTCGCTTGCAGTTCTTCCACCAA-3'), and V4FOR (5'-GCTGAATTCCACCATGCCTCCCAAGTTCAAG-3') and V4REVcyto (5'-GTGGATCCGCTTTTTCAAGTGCAGGATGAT-3'), GS15FOR (5'-ATAGAATTCGGCACGATGGCGGACT-3') and GS15REVcyto (5'-ATAGGATCCATAAGCTTCCGGTTGTCTTG -3'), to make the EGFP expression vectors encoding truncated human syntaxin13, VAMP3, VAMP4, and GS15, respectively.

### Cell culture and transfection

Chinese hamster ovary (CHO)-K1 or HeLa cells were grown in Dulbecco's modified Eagle's medium (DMEM) supplemented with 10% (v/v) fetal bovine serum (FBS; HyClone, Missisauga, ON) at 37°C and 5% (v/v) CO_2_. Cells were transfected using FuGENE6 transfection reagent following the manufacture's suggested protocol (Roche Applied Science, Laval, QC) or calcium phosphate-DNA precipitation. DNA complexes were incubated on the cells for 18 h with GFP-SNAP23 constructs, 48-72 h with the shRNA SNAP23 construct (Open Biosystems, Huntsville, AL) or 24 h when transfecting with other plasmids. Co-transfection studies were performed at a 10:1 molar ratio of DNA-to-marker DNA, except for FLAG-SNAP23/GFP-syntaxin13 co-transfection where the molar ratio was 1:1. Cells were pretreated for 3 h with 5 μg/mL Brefeldin A (BfA) and BfA was maintained in media during experiments where appropriate.

### Immunofluorescence spreading and transwell migration assays

To monitor spreading, cells were plated onto fibronectin (FN) coated (20 μg/ml) glass coverslips for 1.5 h, washed and fixed with 4% (w/v) paraformaldehyde (w/v)/PBS. Samples were then permeabilized and blocked before staining with primary and secondary antibody, followed by washing and mounting. Samples were imaged using a 40X or 63X (NA 1.4) lens on a Leica DM-IRE2 inverted microscope with a Leica TCS SP2 system (Leica, Heidelberg, Germany). Images were captured and 3 D reconstructions were performed using Leica Confocal Software package.

Spreading was quantified by capturing digitized images using a 40X lens. The images were analyzed by outlining cells stained with rhodamine-phalloidin and measuring ventral cell area in ImageJ software. Increases in ventral cell area after 30 min attachment were considered as lamellipodia extension [16,17]. Migration assays were performed as described elsewhere [14]. Statistical comparisons between treatment groups were performed using Student's t-test.

For live cell imaging, CHO cells were transfected, lifted in serum- and phenol red-free DMEM/F12 (Gibco, Missisauga, ON), supplemented with 15 mM HEPES, pH 7.4, plated on FN coated coverslips inserted in a Series 40 chamber in a QE-1 heated platform (Harvard Apparatus Canada, Saint-Laurent, QC), sealed on top with a coverslip and mounted on the microscope stage. Temperature was maintained at 37°C with a TC-324B temperature controller (Harvard Apparatus Canada, Saint-Laurent, QC) for 90 min. Time series were imported into ImageJ software for the generation of kymographs and analysis.

### Integrin and transferrin trafficking assays

To monitor integrin exocytosis, cells were incubated in β1 integrin antibody (AB1950 'fibronectin receptor', Chemicon, Temecula, CA) diluted 1:100 in DMEM for 3 h (internalization), washed with ice-cold PBS and then lifted with trypsin-EDTA (removing surface-bound antibody). Cells were plated on FN-coated coverslips in serum-free DMEM, incubated at 37°C for 10, 20 or 45 min, fixed, and then stained with labeled secondary antibody, with and without permeablization. To monitor integrin localization, transfected CHO cells were incubated with AIIB2 antibody (developed by C.H. Damsky, obtained from the Developmental Studies Hybridoma Bank, Iowa City, IA) diluted 1:75 in DMEM for 1-2 h (internalization). Cells were then quickly washed in cold 0.2 M glycine, pH 2.5, to remove surface label and washed several times with PBS before fixing at 4°C with 4% paraformaldehyde/PBS. For transferrin trafficking assays, transfected CHO cells were grown on coverslips, serum-starved for 1 h and then incubated with 30 μg/mL rhodamine-labeled holo-transferrin (Invitrogen, Mississauga, ON). Cells were quickly washed with cold 0.2 M glycine, pH 2.5, PBS and fixed at 4°C with 4% paraformaldehyde/PBS. Samples were counterstained where appropriate and imaged as outlined above. Integrin endoyctosis/exocytosis was quantified by analyzing integrin fluorescence staining intensities from micrographs in ImageJ software.

### Immunoprecipitation and Western blotting

CHO cells were pretreated for 10 min with 1 mM *N*-ethylmaleimide (NEM) in PBS on ice followed by quenching with 2 mM DTT for 10 min on ice, or treated with 1 mM NEM with 2 mM DTT in PBS for 20 min on ice. Then the cells were lifted and held in suspension for 20 min in DMEM. CHO cells were also lifted and plated on 20 μg/mL FN for 20 min. Cells were harvested and lysed in 50 mM Tris-HCl, pH 7.5, 150 mM NaCl, 1% NP-40, 0.5% sodium deoxycholate, and Sigma complete protease inhibitor cocktail. Lysates were cleared and 10 μg of supernatants were reserved to run as control lysate lanes in Western blots. Of the remaining supernatant, 1-1.5 mg of protein at a concentration of 1-1.5 mg/mL were incubated with syntaxin13 (Stressgen, San Diege, CA), VAMP3 (Affinity Bioreagents, Golden, CO), GS15 (BD Biosciences, Mississauga, ON), SNAP23 (Sigma, Oakville, ON) or GFP (Abcam, Cambridge, MA) antibodies immobilized on cyanogen bromide-activated Sepharose 4B beads (Sigma, Oakville, ON) for overnight. The beads were washed twice with lysis buffer, high salt buffer (50 mM Tris-HCl, pH 7.5, 500 mM NaCl, 0.1% NP-40, 0.05% sodium deoxycholate) and low salt buffer (50 mM Tris-HCl, pH 7.5, 0.1% NP-40, 0.05% sodium deoxycholate) before elution with hot 2X Lammelli buffer for Western blot analysis. The same antibodies used to precipitate SNAREs were used as primary antibodies during Western blotting. Antibody complexes were detected by ECL Plus chemiluminescence (GE Healthcare, Baie d'Urfe QC). Where appropriate membranes were stripped (62.5 mM Tris-HCl, pH 6.8, 2% SDS and 100 mM β-mercaptoethanol) for 15 min at 50°C, washed extensively and reprobed. Densitometry was performed by importing scanned images into ImageJ software and analysis with the 'Gel Analysis' tool pack.

Sequential immunoprecipitates were performed as above except that after adsorption of lysates to immobilized syntaxin13 antibodies for 4 h, the beads were washed three times with lysis buffer and eluted with 100 μL 0.2 M glycine, pH 2.5. The pH of eluate was raised to 7.5 with 1 M Tris, pH 8.5 and a sample was set aside for the detection of VAMP3 and syntaxin13 before the rest was diluted to one milliliter with lysis buffer. Then the diluted elute was adsorbed to immobilized VAMP3 antibodies for 4 h. The beads were washed twice with lysis buffer before elution with hot 2X Lamelli buffer and analysis by Western blot. Membranes were blotted for SNAP23.

### Quantitative ruffling assay

Cells were plated on FN-coated (10 μg/ml) glass coverslips for 3 h in serum-free DMEM. The cells were then treated with 500 nM PMA in DMEM, or DMEM alone as control, for 10 min. Cells were fixed with 4% paraformaldehyde/PBS and stained with rhodamine-phalloidin (Invitrogen, Mississauga, ON). An automated method to detect and quantify F-actin-containing ruffles in confocal micrographs was used and this approach is described in detail elsewhere [31].

## Abbreviations

CHO: Chinese hamster ovary; DMEM: Dulbecco's modified Eagle's medium; ECM: extracellular matrix; FBS: fetal bovine serum; FN: fibronectin; GFP: green fluorescent protein; NSF: *N*-ethylmeimide-sensitive [NEM] fusion protein; SNARE: soluble NSF attachment protein receptor; TeTx-LC: tetanus toxin light chain; TGN: trans-Golgi network; VAMP: vesicle associate membrane protein.

## Authors' contributions

MS conceived and carried out the immunoprecipitation, lammellipodia extension and immunofluorescence trafficking assays, and drafted the initial manuscript. QY conceived and performed the ruffling assays. MJK carried out the migration assays and contributed to the ruffling assays. DWM assisted in the immunoprecipitation assays. KCW contributed experimentally to the shRNA knock-down studies. AB generated several of the SNARE constructs and contributed to the spreading assays. MGC conceived/designed the study, analyzed/processed data, assembled figures and edited the manuscript. All authors read and approved the final manuscript.

## Supplementary Material

Additional file 1 **Quantification of SNAREs**. (A and B) CHO-K1 cells were held in suspension after pretreatment with 1 mM NEM and 2 mM DTT (*Susp*), held in suspension after pretreatment with 1 mM NEM (*Susp+NEM*) or plated on FN (*FN*) for 20 min. (A) Graph shows the intensities of syntaxin13 bands from SNAP23 immunoprecipitates, normalized to samples adherent to FN (mean +/- SD of at least three experiments) (B) Intensities of syntaxin13 bands from VAMP4 immunoprecipitates, normalized to samples adherent to FN (mean +/- SD of at least three experiments). (C and D) CHO-K1 cells were treated with 500 nM PMA in DMEM (*PMA*) or DMEM alone (*control*) for 10 mins, lysed and VAMP4 (C) or VAMP3 (D) were immunoprecipitated. Graphs show intensities of SNAP23 bands from VAMP4 immunoprecipitates (C) or VAMP3 immunoprecipitates (D), normalized to non-induced samples. Data represent means +/-SD from at least 3 independent experiments. Click here for file

Additional file 2 **Complex containing Syntaxin13 and SNAP23 does not form after lysis of cells**. Cells transfected with FLAG-SNAP23 or GFP-syntaxin13 alone were held in suspension and lysed. After lysis, the FLAG-SNAP23-containing and GFP-syntaxin13-containing lysates were mixed for 1 h (*In Vitro Mix*) followed by immunoprecipitation with anti-GFP antibody overnight. Cotransfected cells were held in suspension (*Susp*) or plated on FN (*FN*), lysed and then immunoprecipitations were done with anti-GFP antibody. GFP immunoprecipitates were probed for the presence of FLAG-tagged SNAP23, *upper blot*, using anti-FLAG antibody, and the same membrane was reprobed for GFP-syntaxin13, *lower blot*, using anti-GFP antibody. "*Bead+Lys*" is eluate from sepharose beads incubated with lysate and "*GFP Ab*" is eluate from sepharose-coupled anti-GFP in lysis buffer. Click here for file

Additional file 3 **Dominant-negative SNAREs bind cognate SNAREs and impair endogenous binding**. CHO-K1 cells were transiently transfected with truncated GFP-tagged constructs of syntaxin13 (syn13cyto-GFP), VAMP3 (VAMP3cyto-GFP), VAMP4 (VAMP4cyto-GFP) or SNAP23 (SNAP23^CΔ9^-GFP), lysed and cognate SNAREs were immunoprecipitated with antibodies coupled to sepharose beads. Immunoprecipitates were washed and analyzed by SDS-PAGE-western blotting for inhibitor and endogenous SNARE binding. (A) Syntaxin13 western blot of SNAP23 immunoprecipitaes from syn13cyto transfected cells. (B) VAMP3 western blot of SNAP23 and syntaxin13 immunoprecipitaes from VAMP3cyto transfected cells. (C) VAMP4 western blot of SNAP23 and syntaxin13 immunoprecipitaes from VAMP4cyto transfected cells. (D) SNAP23 western blot of VAMP3 and syntaxin13 immunoprecipitaes from SNAP23^CΔ9 ^transfected cells. Click here for file

Additional file 4 **In cells expressing dominant-negative SNARE domains integrin co-localizes with Rab4**. CHO-K1 cells were transiently transfected for 22 h with GFP [A-D] or truncated GFP-tagged constructs of VAMP3 (VAMP3cyto) [E-H], syntaxin13 (syntaxin13cyto) [I-L], VAMP4 (VAMP4cyto) [Q-T] or 15 h with SNAP23 (SNAP23^CΔ9^)[M-P]. Cells were labeled with anti-β1 integrin antibody in serum free media for 1 h to allow internalization of the label. Cells were then washed in 0.2 M glycine, pH 2.5, fixed and stained for the labeled integrin [B, F, J, N, R] and Rab4 [C, G, K, O, S]. Overlays of the Rab4 and β1 integrin staining are shown in D, H, L, P and T. Images are 3 D reconstructions of a z-series. Scale bar represents 10 μm. Click here for file

Additional file 5 **Dominant-negative SNARE domains inhibit trafficking of transferrin into a perinuclear compartment**. CHO-K1 cells were transiently transfected for 22 h with truncated GFP-tagged constructs of VAMP3 (VAMP3-cyto) [A], syntaxin13 (syntaxin13-cyto) [C], VAMP4 (VAMP4-cyto) [G], or 15 h with SNAP23 (SNAP23^CΔ9^) [E]. To load the perinuclear recycling compartment with Tfn, cells were serum starved for 1 hr followed by incubation with 30 μg/mL rhodamine-Tfn in serum-free media for 15 min. Coverslips were briefly washed with 0.2 M glycine, pH 2.5, to remove surface bound Tfn, washed three times with PBS and fixed in 4% paraformaldehyde in PBS. Arrows in B, D and F point to perinuclear, rhodamine-Tfn-containing compartment in non-transfected control cells. Images are 3 D reconstructions of a z-series. Scale bar represents 10 μm Click here for file
