# Supplementary material for: Lamellipodium extension and membrane ruffling require different SNARE-mediated trafficking pathways
Source: BMC Cell Biol. 2010 Aug 10;11:62. doi: 10.1186/1471-2121-11-62 (PMC2925818; doi:10.1186/1471-2121-11-62)
Supplement: Additional file 1 — Quantification of SNAREs. (A and B) CHO-K1 cells were held in suspension after pretreatment with 1 mM NEM and 2 mM DTT (Susp), held in suspension after pretreatment with 1 mM NEM (Susp+NEM) or plated on FN (FN) for 20 min. (A) Graph shows the intensities of syntaxin13 bands from SNAP23 immunoprecipitates, normalized to samples adherent to FN (mean +/- SD of at least three experiments) (B) Intensities of syntaxin13 bands from VAMP4 immunoprecipitates, normalized to samples adherent to FN (mean +/- SD of at least three experiments). (C and D) CHO-K1 cells were treated with 500 nM PMA in DMEM (PMA) or DMEM alone (control) for 10 mins, lysed and VAMP4 (C) or VAMP3 (D) were immunoprecipitated. Graphs show intensities of SNAP23 bands from VAMP4 immunoprecipitates (C) or VAMP3 immunoprecipitates (D), normalized to non-induced samples. Data represent means +/-SD from at least 3 independent experiments. [file 1471-2121-11-62-S1.PDF]

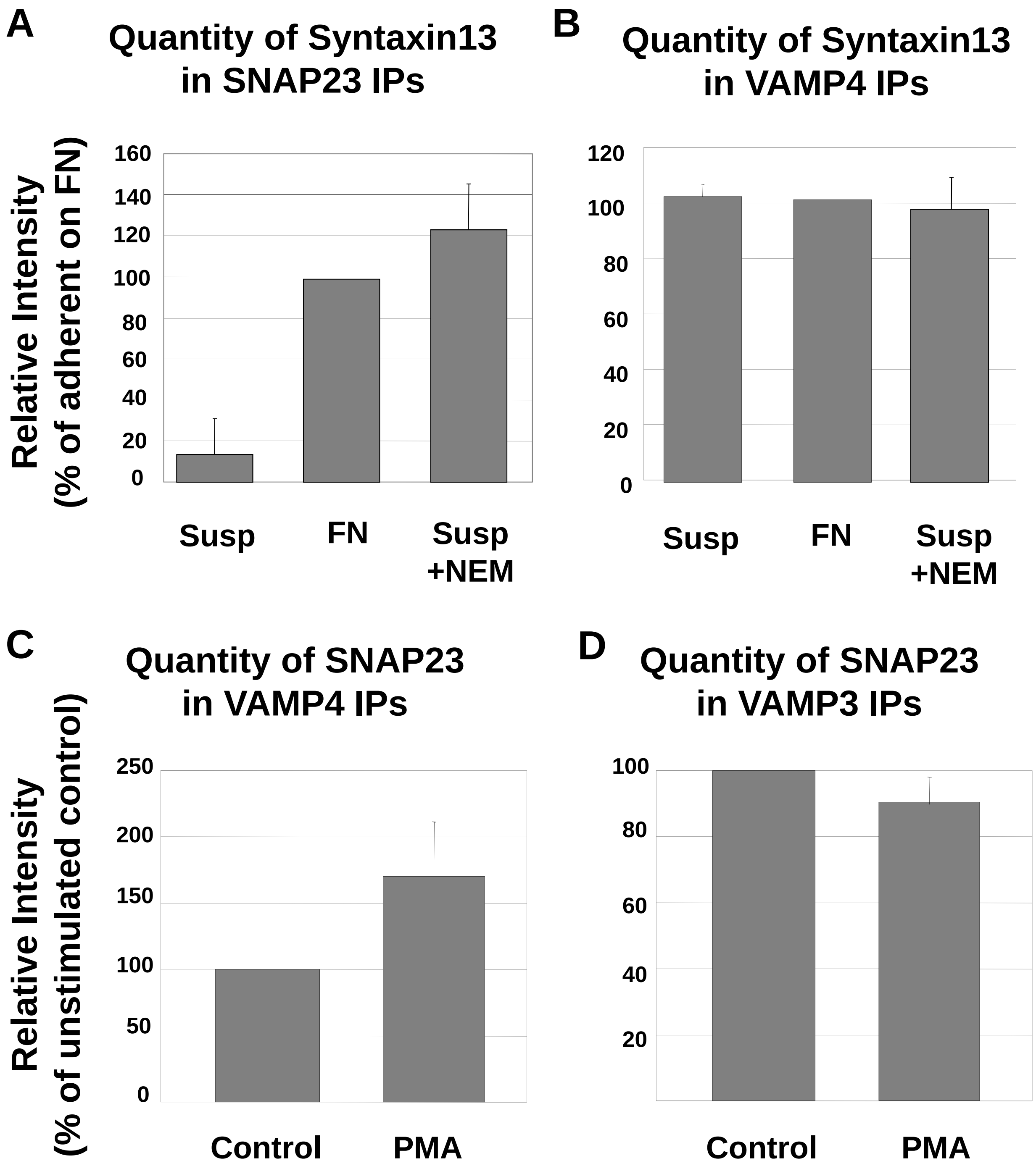
A
Quantity of Syntaxin13
in SNAP23 IPs
Relative Intensity
(% of adherent on FN)
160
140
120
100
80
60
40
20
0
Susp
FN
Susp
+NEM
B
Quantity of Syntaxin13
in VAMP4 IPs
120
100
80
60
40
20
0
Susp
FN
Susp
+NEM
C
Quantity of SNAP23
in VAMP4 IPs
Relative Intensity
(% of unstimulated control)
250
200
150
100
50
0
Control
PMA
D
Quantity of SNAP23
in VAMP3 IPs
100
80
60
40
20
Control
PMA
