# Supplementary figures and images for: Lamellipodium extension and membrane ruffling require different SNARE-mediated trafficking pathways
Source: BMC Cell Biol. 2010 Aug 10;11:62. doi: 10.1186/1471-2121-11-62 (PMC2925818; doi:10.1186/1471-2121-11-62)

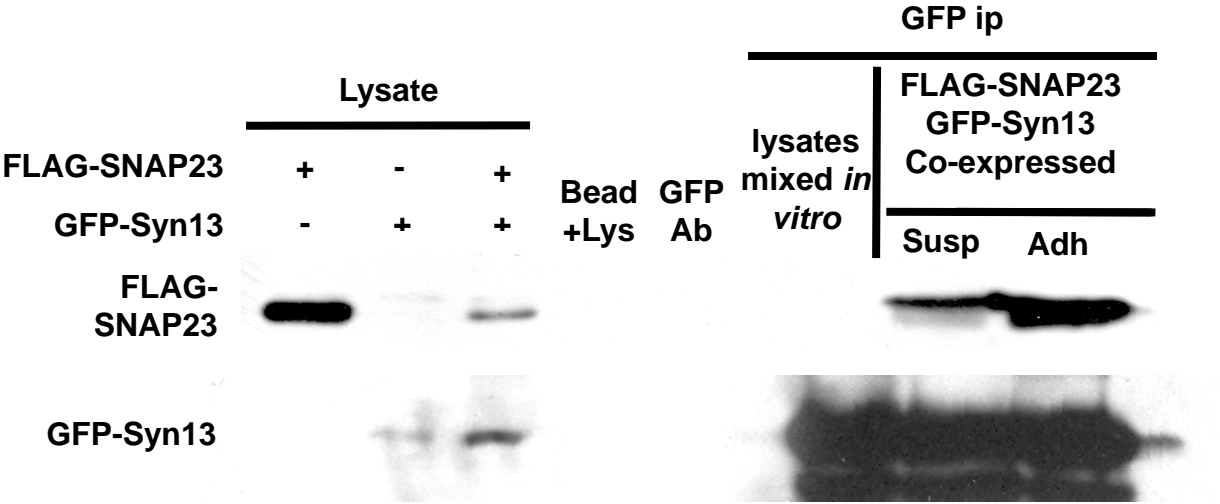

Supplement: Additional file 2 — Complex containing Syntaxin13 and SNAP23 does not form after lysis of cells. Cells transfected with FLAG-SNAP23 or GFP-syntaxin13 alone were held in suspension and lysed. After lysis, the FLAG-SNAP23-containing and GFP-syntaxin13-containing lysates were mixed for 1 h (In Vitro Mix) followed by immunoprecipitation with anti-GFP antibody overnight. Cotransfected cells were held in suspension (Susp) or plated on FN (FN), lysed and then immunoprecipitations were done with anti-GFP antibody. GFP immunoprecipitates were probed for the presence of FLAG-tagged SNAP23, upper blot, using anti-FLAG antibody, and the same membrane was reprobed for GFP-syntaxin13, lower blot, using anti-GFP antibody. "Bead+Lys" is eluate from sepharose beads incubated with lysate and "GFP Ab" is eluate from sepharose-coupled anti-GFP in lysis buffer. [file 1471-2121-11-62-S2.PDF]

**A**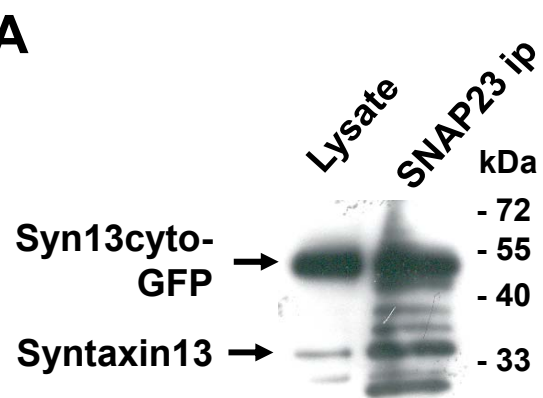**B**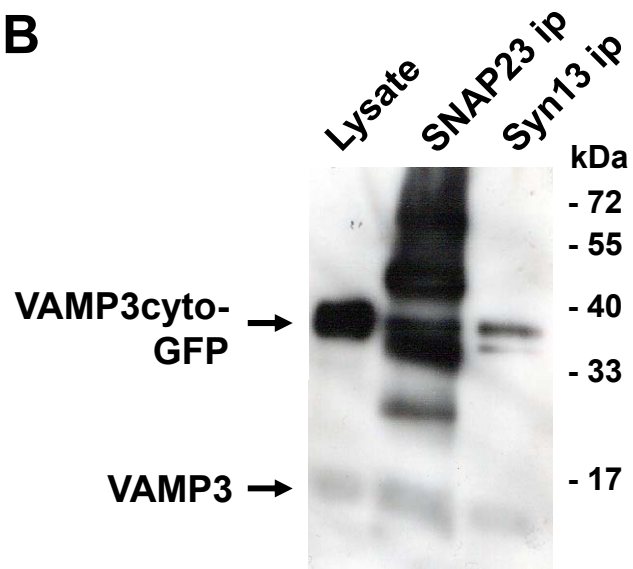**C**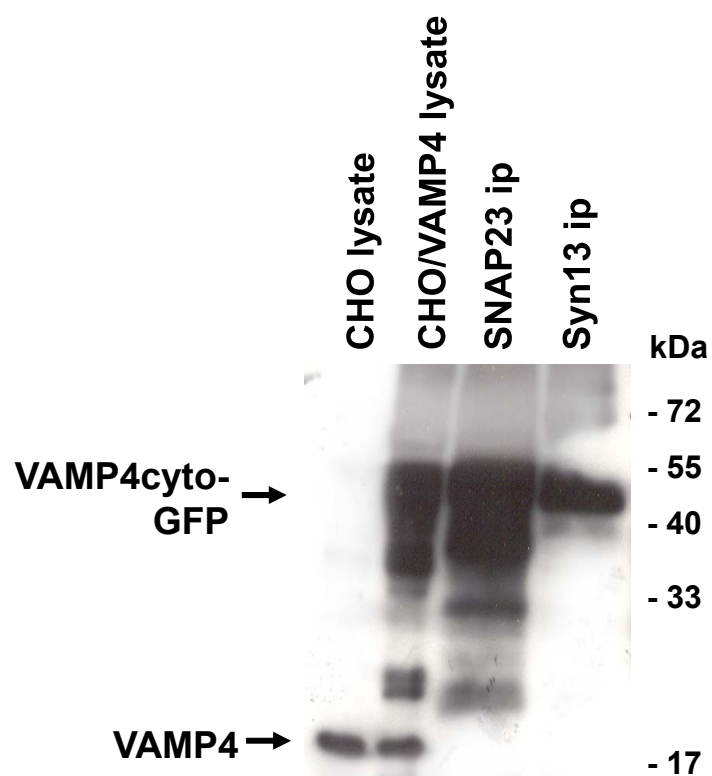**D**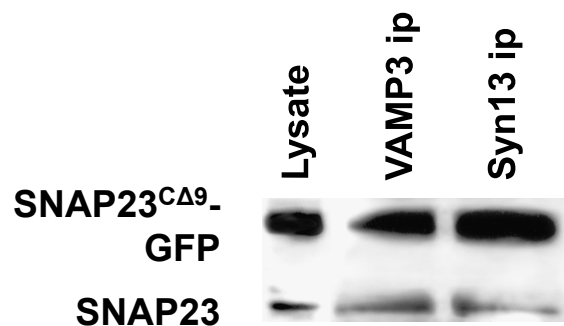

Supplement: Additional file 3 — Dominant-negative SNAREs bind cognate SNAREs and impair endogenous binding. CHO-K1 cells were transiently transfected with truncated GFP-tagged constructs of syntaxin13 (syn13cyto-GFP), VAMP3 (VAMP3cyto-GFP), VAMP4 (VAMP4cyto-GFP) or SNAP23 (SNAP23CΔ9-GFP), lysed and cognate SNAREs were immunoprecipitated with antibodies coupled to sepharose beads. Immunoprecipitates were washed and analyzed by SDS-PAGE-western blotting for inhibitor and endogenous SNARE binding. (A) Syntaxin13 western blot of SNAP23 immunoprecipitaes from syn13cyto transfected cells. (B) VAMP3 western blot of SNAP23 and syntaxin13 immunoprecipitaes from VAMP3cyto transfected cells. (C) VAMP4 western blot of SNAP23 and syntaxin13 immunoprecipitaes from VAMP4cyto transfected cells. (D) SNAP23 western blot of VAMP3 and syntaxin13 immunoprecipitaes from SNAP23CΔ9 transfected cells. [file 1471-2121-11-62-S3.PDF]

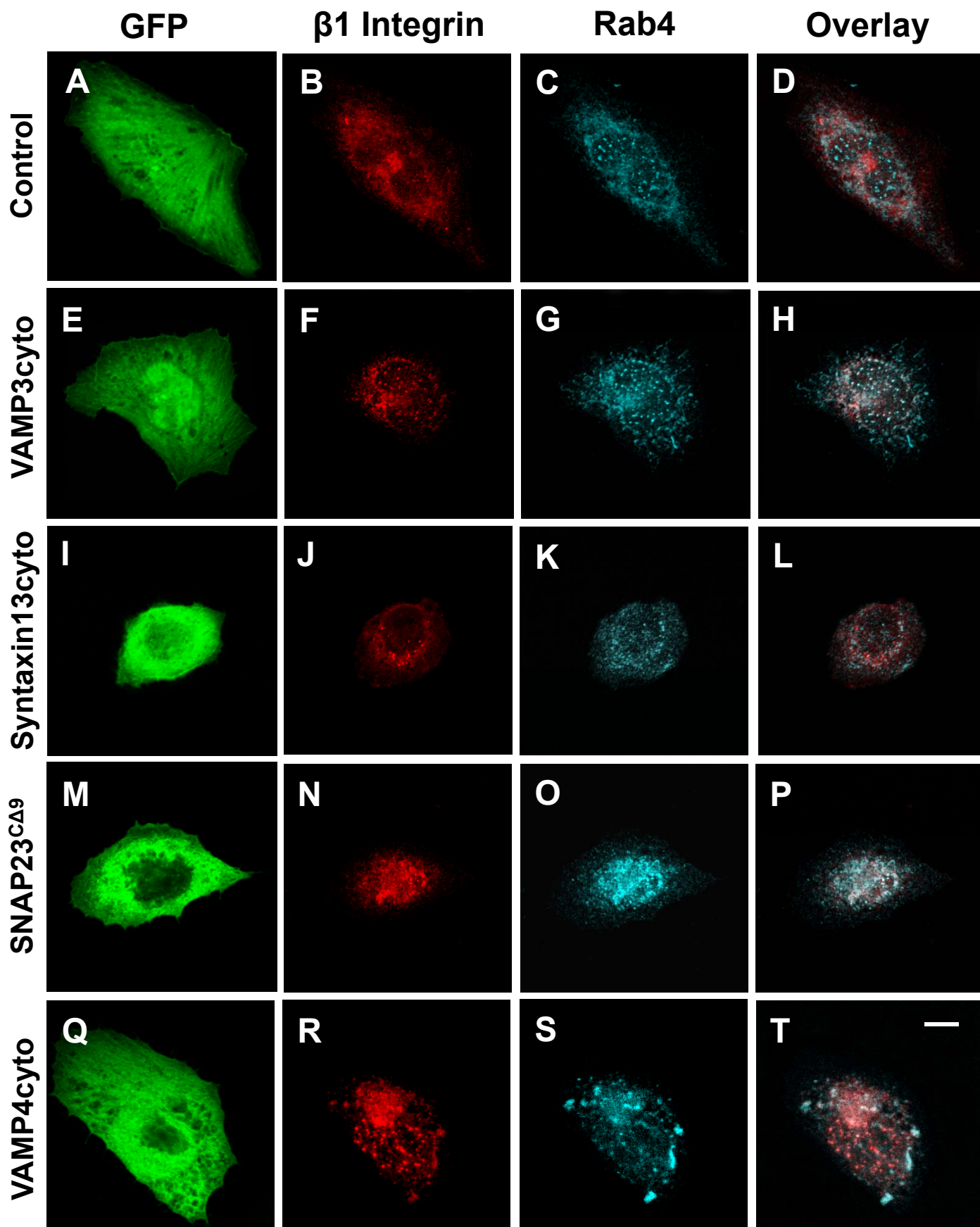

Supplement: Additional file 4 — In cells expressing dominant-negative SNARE domains integrin co-localizes with Rab4. CHO-K1 cells were transiently transfected for 22 h with GFP [A-D] or truncated GFP-tagged constructs of VAMP3 (VAMP3cyto) [E-H], syntaxin13 (syntaxin13cyto) [I-L], VAMP4 (VAMP4cyto) [Q-T] or 15 h with SNAP23 (SNAP23CΔ9)[M-P]. Cells were labeled with anti-β1 integrin antibody in serum free media for 1 h to allow internalization of the label. Cells were then washed in 0.2 M glycine, pH 2.5, fixed and stained for the labeled integrin [B, F, J, N, R] and Rab4 [C, G, K, O, S]. Overlays of the Rab4 and β1 integrin staining are shown in D, H, L, P and T. Images are 3 D reconstructions of a z-series. Scale bar represents 10 μm. [file 1471-2121-11-62-S4.PDF]

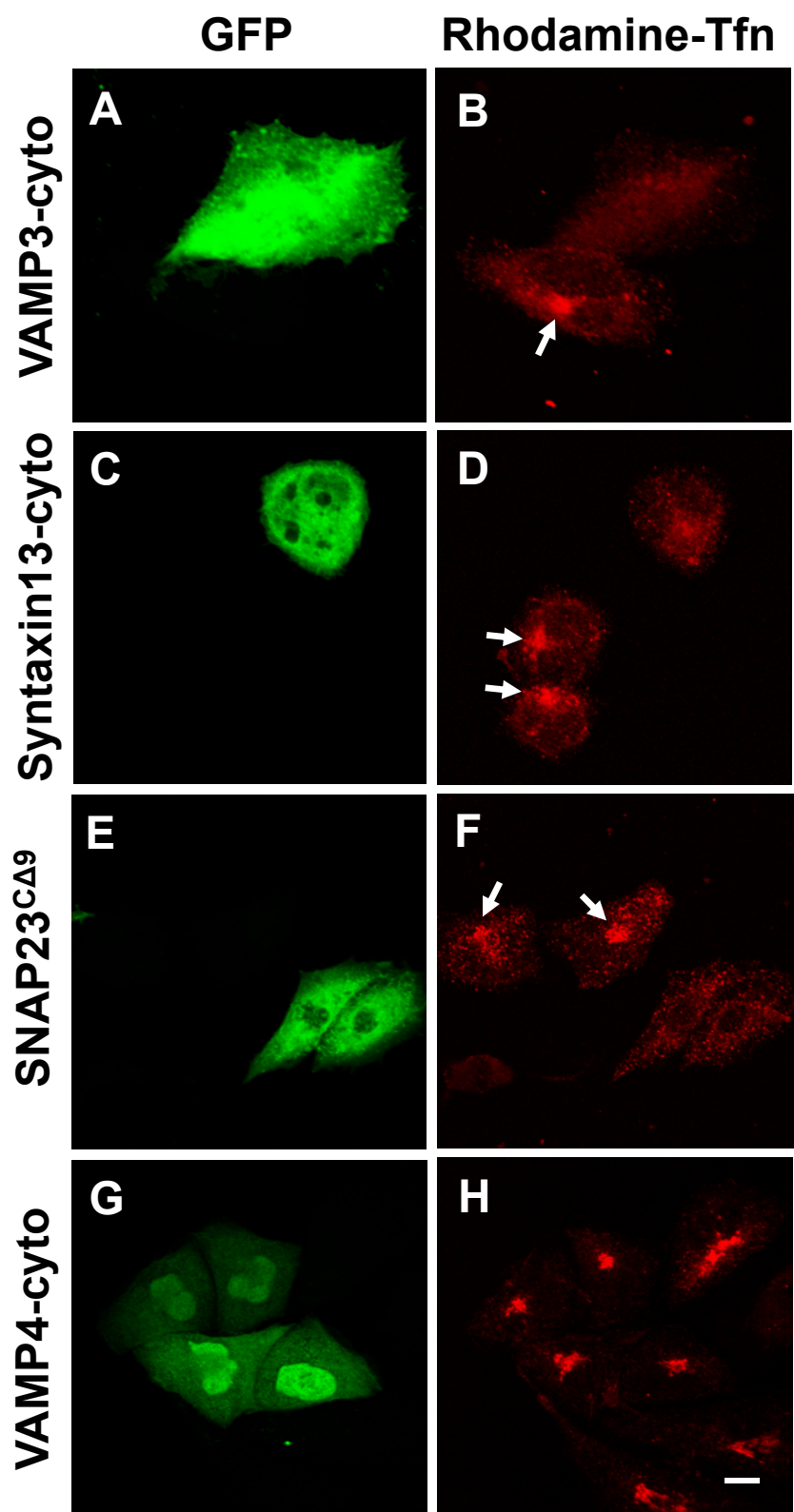

Supplement: Additional file 5 — Dominant-negative SNARE domains inhibit trafficking of transferrin into a perinuclear compartment. CHO-K1 cells were transiently transfected for 22 h with truncated GFP-tagged constructs of VAMP3 (VAMP3-cyto) [A], syntaxin13 (syntaxin13-cyto) [C], VAMP4 (VAMP4-cyto) [G], or 15 h with SNAP23 (SNAP23CΔ9) [E]. To load the perinuclear recycling compartment with Tfn, cells were serum starved for 1 hr followed by incubation with 30 μg/mL rhodamine-Tfn in serum-free media for 15 min. Coverslips were briefly washed with 0.2 M glycine, pH 2.5, to remove surface bound Tfn, washed three times with PBS and fixed in 4% paraformaldehyde in PBS. Arrows in B, D and F point to perinuclear, rhodamine-Tfn-containing compartment in non-transfected control cells. Images are 3 D reconstructions of a z-series. Scale bar represents 10 μm [file 1471-2121-11-62-S5.PDF]
